# A Framework of Vertebra Segmentation Using the Active Shape Model-Based Approach

**DOI:** 10.1155/2011/621905

**Published:** 2011-07-31

**Authors:** Mohammed Benjelloun, Saïd Mahmoudi, Fabian Lecron

**Affiliations:** Faculty of Engineering, University of Mons, Biosys Pole, Place du Parc, 20-7000 Mons, Belgium

## Abstract

We propose a medical image segmentation approach based on the Active Shape Model theory. We apply this method for cervical vertebra detection. The main advantage of this approach is the application of a statistical model created after a training stage. Thus, the knowledge and interaction of the domain expert intervene in this approach. Our application allows the use of two different models, that is, a global one (with several vertebrae) and a local one (with a single vertebra). Two modes of segmentation are also proposed: manual and semiautomatic. For the manual mode, only two points are selected by the user on a given image. The first point needs to be close to the lower anterior corner of the last vertebra and the second near the upper anterior corner of the first vertebra. These two points are required to initialize the segmentation process. We propose to use the Harris corner detector combined with three successive filters to carry out the semiautomatic process. The results obtained on a large set of X-ray images are very promising.

## 1. Introduction

In some circumstances, it is not easy for humans to distinguish objects in X-ray images from their background. Developing algorithms and methods for obtaining a proper object extraction is one of the most important research topics in the image processing field. Computer-based image segmentation facilitates the domain expert work and can automate tasks dealing with interpretation of medical images. 

In this paper, we focus on vertebra segmentation applied to X-ray images. This operation is generally the first step to be performed before any disease diagnosis or vertebral mobility analysis. Therefore, this segmentation process is an essential and critical task. Indeed, the segmentation should be effective enough in order to analyze the mobility of the spinal column and accurately estimate the movement of each vertebra.

The goal of the segmentation process is to exploit only the useful information for image interpretation. A wide variety of techniques and approaches have been proposed in the literature. We can cite active contours (or snake) which present a powerful method for edge extraction of objects having arbitrary shapes [1–3]. This approach has been investigated and applied in various ways in [4–6]. Another widely used approach is the level set-based methods which is a variation of the active contours approaches, [7]. 

These two methods have recently been used as new paradigms for a large number of segmentation methods due to their flexibility to deform the shape that must be detected. Nevertheless, such methods have an inherent limitation that makes them nonsuitable for many medical segmentation tasks where an *a priori* knowledge about the shape to be segmented is required, and also when an initialization too close to the shape to be segmented is needed.

In related works on medical images analysis, Luo [8] introduced an automated medical image segmentation algorithm used to locate volumetric objects such as brain tumors in Magnetic Resonance Imaging (MRI) images. In his work, the author proposed an algorithm which deals with MRI slices as a three-dimensional (3D) object. All the processes of segmentation are done in a 3D space. Firstly, it removes noisy voxels with 3D nonlinear anisotropic filtering. Secondly, it uses a novel deformable surface model to segment an object from the MRI. A dynamic gradient vector flow was used to form the surface model. Experiments have been done on segmenting tumors from real MRI data of the human head. This algorithm reports accurate 3D tumor segmentation.

Other segmentation methods are the template-matching approaches. These methods are used to identify simple geometric shapes like ellipses or parabolas in an image. They match a predefined template to the location of some extracted features such as image gradient, boundary points, or grey level value. These techniques are specific to the structure of segmentation. They can be easily implemented and can give effective results when an appropriate model is chosen [9, 10]. Other methods based on the Hough Transform algorithm were applied to vertebrae detection field in [11, 12]. The model-based segmentation approaches, such as those employing Active Shape Models (ASMs), use a statistical shape models (SSMs), to identify specified forms in an image. They were introduced by Cootes et al. in [13] and have been proven in recent years to be very useful for medical image segmentation. We propose to use this method for vertebra segmentation in X-ray images.

Active Shape Model (ASM) [14] is described by the statistical shape model of objects. This method is used to extract shapes from images. The algorithm deforms an initial shape repeatedly in order to fit a variant of the statistical shape model also named Point Distribution Model (PDM), to an object in a new image. Shapes are constrained by the Point Distribution Model. Main variation modes are used to compute the variation of the mean shape. A subarea of possible forms for the object is created. The average shape is then selected and used to initialize the search of an object in a new image.

This method is commonly used for MRI image segmentation in the brain area or for cardiac images. However, the quality of the segmentation is highly dependent on the initialization phase. A good initialization is required to accelerate and help the morphing phase to obtain effective results. The ASM relies on the fact that the search is based on an *a priori* knowledge of the target object. This is an important behavior of this technique as it allows the user to choose the images and to carefully place the “landmarks” for the creation of a model. The domain expert knowledge can be used in such tasks.

In this context, Rueda et al. [15] propose an Active-Shape-Model-based method which is guided by the strategy of equalization of the variance contained in a training set for selecting landmarks. In their work, the chosen landmarks are positioned around each contour in such a manner to equally distribute the total variance existing in the training set.

Another variation of the ASM method is Active Appearance Model (AAM) which is largely described in the scientific literature [16]. However, the ASM method provides better and faster results [14]. Moreover, the method depends on a few parameters. It is not the case of the other deformable model-based methods like snakes. But ASM, as snakes methods, is sensitive to the accuracy of the initialization phase. Thus, it is highly important to improve this phase. ASM was tested and approved in several medical applications: knees, volumes of brain, thoracic cage, and even faces [14, 17].

Several publications [18–22] propose different methods to extract vertebra contours from X-ray images, like polar signature, template matching, active contours, and Discrete Dynamic Contour Model or Harris Detector.

In this paper, we propose to use an Active Shape Model segmentation approach in order to extract vertebra contours. In addition, we focus on improving the initialization phase of this method. Therefore, we propose a semiautomatic method allowing to ideally place the mean shape on the vertebrae to be segmented. We achieve this task by using the Harris corner detector followed by a series of filters aiming to detect the two anterior corners of each vertebra on the X-ray image.

The structure of the paper is presented as follows: in Section 2 we present an overview of the different steps related to the segmentation method proposed. Those steps are as follows: learning, model design, initialization, and segmentation. In Section 3, we present the experimental results, including the study of the influence of a set of parameters on the segmentation results, such as the number of sample images, the number of landmarks per vertebra, the profile structure, and the type of model used for the segmentation. Finally, we give a general conclusion in Section 4.

## 2. Method Overview

 In this paper we propose a segmentation approach based on Active Shape Model in order to identify vertebra edges. This method allows to model vertebrae whose appearance and location in the spinal column differ depending on the patient.

The statistical nature of the method involves the use of sample shapes that can be adopted by the object model. The sample must be as representative as possible to improve the quality of the model. In fact, the ASM algorithm defines a set of forms that well characterize the shape to be identified. This set of shapes that contains the different variations of the mean shape depends on the sample. Therefore, if the created model is not realistic enough, it could accept some shapes that are not really corresponding to the desired shape or conversely reject the shapes that are good. This aspect is the first difficulty of the ASM-segmentation-based method. It is important to know or to estimate as precisely as possible the actual distribution of the shapes to model.

Once the model is determined, it can be used to detect other similar shapes in new images. To this end, the mean shape model is extracted and placed in an area of interest. The shape is then iteratively warped until it fits at best the real edge of the object.

**Figure 1:**
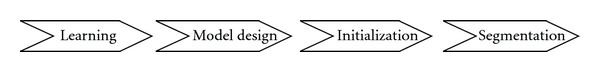
The steps of the ASM framework.

The ASM method [13] is composed of 4 steps (Figure 1).

**(1) Learning**

it consists of the placement of landmarks on the images in order to describe the vertebrae. The specialist knowledge can be included in this step.

**(2) Model Design**

all the marked shapes have to be aligned before the creation of the model. It could be useful for the specialist to build a model corresponding to a particular pathology. For instance, if he wishes to detect vertebra arthritis, the vertebrae of the sample is presented as a shade whiter than normal and shows an abnormal bone growths. Once the model is created, these same patterns can be found in an X-Ray with this disease.

**(3) Initialization**

placing the mean shape model on the area of interest. This step can be manual or semiautomatic.

**(4) Segmentation**

each point of the mean shape evolves in order to fit the vertebra edge.

### 2.1. Learning

The goal of the learning phase is to build an image sample which will be the basis for the model creation. An annotated training set is used to build this model [13]. The training set comes from hand annotation or semiautomatic segmentation of a set of training images, followed by manual or automatic landmarking methods to describe the surface. By analyzing shape variations over the training set, the model containing these variations can be built. Therefore, each vertebra must be described by landmarks. These particular points have to be identifiable in any shape. It is also necessary to specify the number of landmarks per vertebra to be considered during the annotation phase.

**Figure 2:**
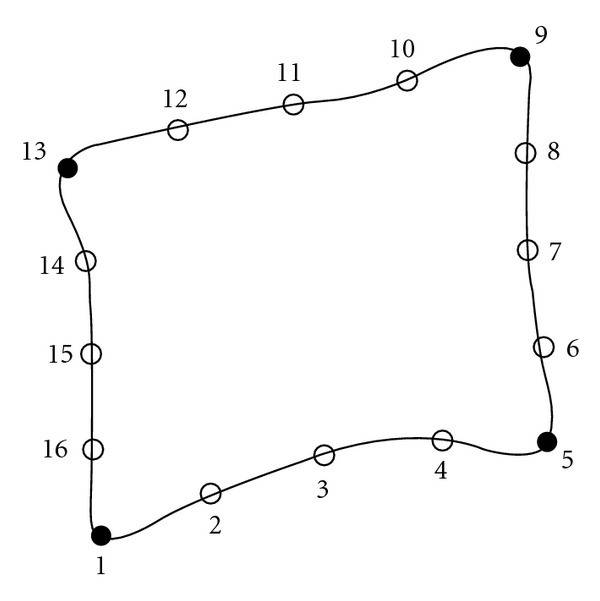
Vertebra marking.

It is a common practice to choose as landmarks the corners of the vertebra and a reasonable number of equidistant points between the corners.  Figure 2 shows an example of vertebra marking. Points 1, 5, 9, and 13 identify the corners while others are scattered along the edges.

The shape of an object is represented by a set of *n* points located on its surface. It is represented by a vector *x*_*i*_, defined as the juxtaposition of the coordinates of each point of reference. The variable *n* represents the number of landmarks. Naturally, a greater *n* improves the quality of the results but increases also the computing time:

(1) $$x_{i}={\left(x_{i1},y_{i1};x_{i2},y_{i2};\ldots ;x_{ik},y_{ik};\ldots ;x_{in},y_{in}\right)}^{T}.$$

This marking phase is time-consuming as the specialist has to put the landmarks manually on the images. He can then determine the location of strategic points that will be used in the model. Furthermore, automated tools such as polygonal approximation can be considered to achieve this goal. However, a purely automatic marking requires noiseless images or a preidentified contour.

In addition, one can imagine semiautomatic systems where the user could correct the annotation.

### 2.2. Model Design

The annotated shapes are generally positioned at various locations and orientations on vertebra edges. For this reason, it is necessary to align all these shapes in order to make a correct statistical treatment [13].

There are several alignment techniques, but the generalized Procrustes analysis is the most commonly used [14]. In this method, we first consider the alignment of two shapes. This induces the minimization of a weighted sum of distances between equivalent reference points of two forms. To this aim, each of them can undergo a rotation, a translation, and a scaling. The applied algorithm is explained as follow is:

1. align each shape of the sample on the first one;
2. repeat until convergence:
  - compute the mean shape,
  - adjust the mean shape:
    - to a size, an orientation and an origin by default,
    - to the first shape,
  - align each shape on the mean shape.

**Figure 3:**
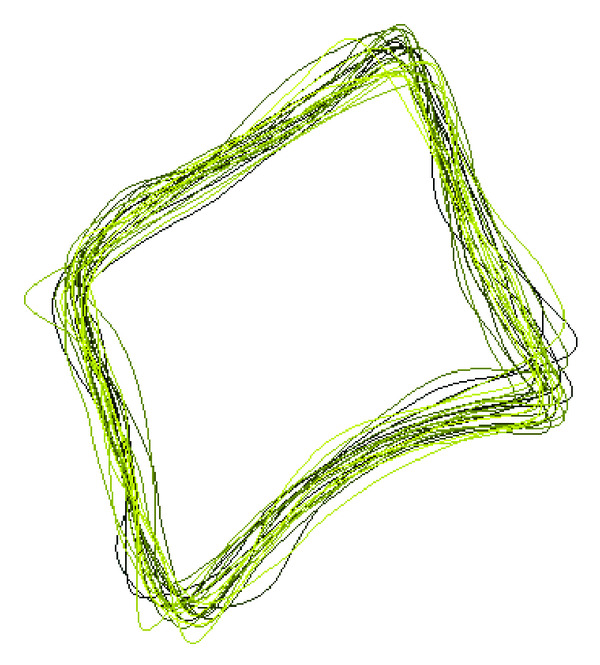
An example of alignment.

The purpose of the iterative process is to reduce the dependency of the model to the first shape. Concerning the adjustment of the mean shape at the second step, we have chosen to align it to the first shape. An example of vertebra alignment is given at Figure 3.

The mean shape is characterized by the arithmetic mean of coordinates describing each element of the sample after the alignment. We have

(2) $$\overset{®}{x}=\frac{1}{f}\overset{f}{\underset{i=1}{\sum }}x_{i},$$

with *f* being the number of shapes used in the training set.

The mean shape constitutes the basis of the vertebra edge detection process. A set of possible models are derived from this mean shape by moving the points through specific directions corresponding to the eigenvectors of the sample variance-covariance matrix, (*p*_*i*_).

The model (see (3)) is defined by the mean shape $\overset{®}{x}$, the matrix *P* of the most significant eigenvectors *p*_*i*_, and a vector of weight factors *b*. We can write

(3) $$x=\overset{®}{x}+Pb,$$

with *P* = (*p*_1_, *p*_2_,…, *p*_*t*_) and *b* = (*b*_1_,*b*_2_,…,*b*_*t*_)^*T*^.

This model is used to decide if an object from an image can be considered as acceptable. As the coordinates of the landmarks of an object are known and as the eigenvectors are unit vectors (*p*_*i*_^*T*^*p* = 1), it is possible to determine the vector *b* by

(4) $$b=P^{T}\left(x-\overset{®}{x}\right).$$

The values of the factors *b*_*i*_ allow to detect if an object is convenient to the model. These values can vary in the following manner [13]:

(5) $$-3\sqrt{\lambda_{i}}\leq b_{i}\leq 3\sqrt{\lambda_{i}},$$

with *λ*_*i*_ being the eigenvalues corresponding to the eigenvectors *p*_*i*_.

### 2.3. Initialization

The search initialization consists of placing the mean shape previously computed on the image as close as possible to the real object. This operation can be done manually or in a semiautomatic way. In a manual initialization, the user is prompted to select the left side of each vertebra by clicking on the left superior and inferior corners. The mean shape is positioned according to this information.

**Figure 4:**
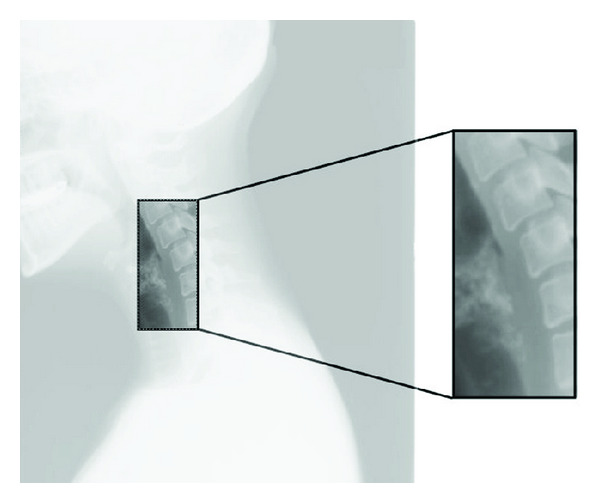
Reduction of the window search.

The semiautomatic initialization does not require more than two clicks to limit the search window, including the left edge of the *N* vertebrae to identify. For this purpose, the user is asked to select the superior left corner of the first vertebra and the inferior left of the last one.  Figure 4 illustrates the type of image that we obtain by applying this image area limitation.

**Figure 5:**
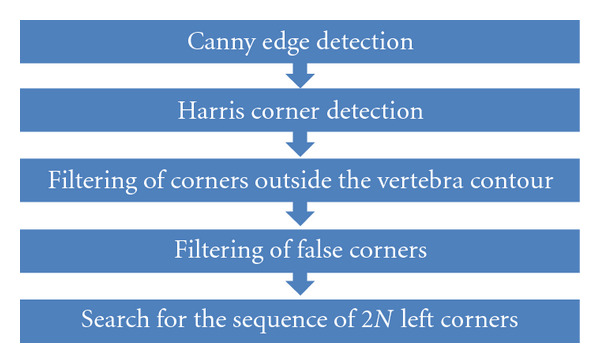
Procedure to detect corners of each vertebra.

In this paper, we propose a set of steps in order to place the mean shape on the vertebrae, in a semiautomatic way.  Figure 5 shows how to detect the superior and inferior left corners of each vertebra.

The Canny filter [23] allows detecting edges in an image by taking advantage of the information given by the intensity gradient. The Harris corner detector is a popular interest point detector proposed by Harris and Stephens [24]. the most advantageous aspect of these detectors is their strong invariance under rotation, scale, illumination variation, and image noise.

**Figure 6:**
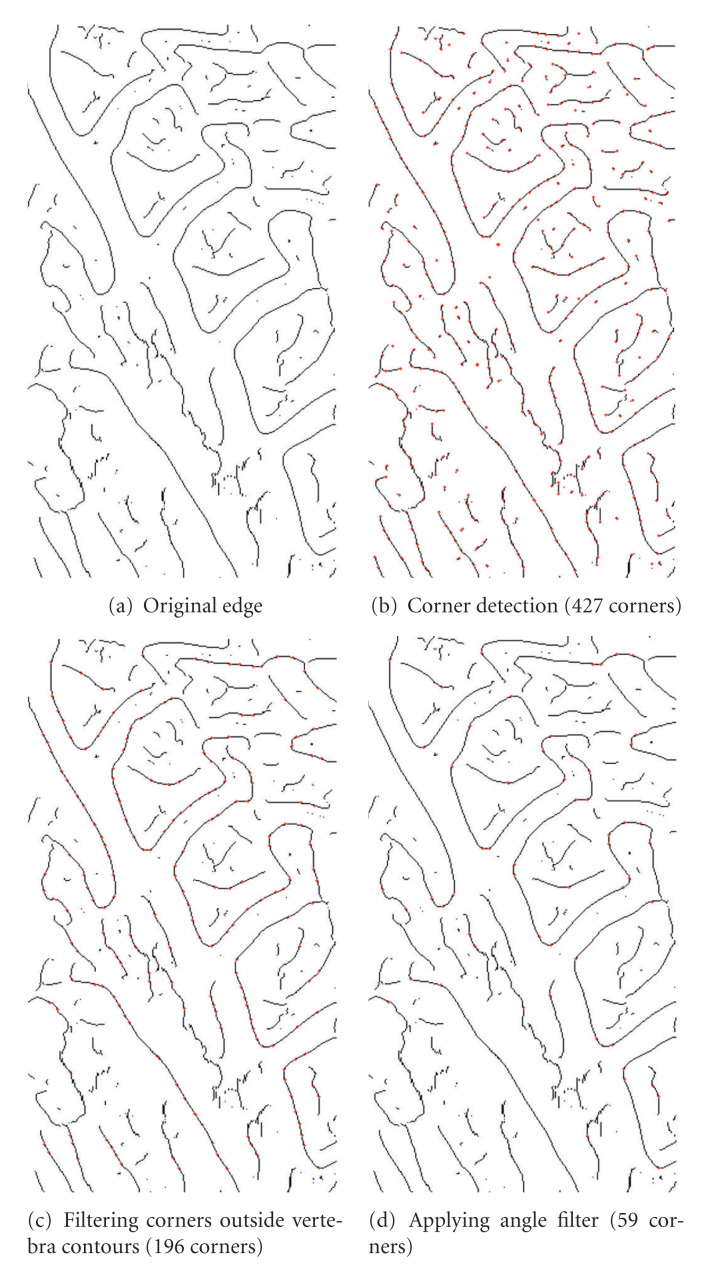
Illustration of the effect of filtering out corners neighboring a contour (b) and the filtering of false corners (c).

However, the Harris detector produces a high number of corners as shown in Figure 6(b). It is important to reduce this number in order to apply the downstream methods. For such process, we will include two filters: the filtering of neighboring corners outside contour and the angle filtering.

Filtering the corners outside the vertebra contour is based on the search for neighboring points. During this process, some points belonging to the Canny edge can be filtered. This occurs when a Harris corner is isolated (e.g., in the case of the extremity of a contour) or when the edge is too small.

When Harris corner is used, if finding good neighbors from a distance equal to an estimated height of a vertebra is not possible, then, the point is eliminated. The effect of this filtering is shown in Figure 6(c). For this example, 231 points on the 427 Harris corners have been deleted.

**Figure 7:**
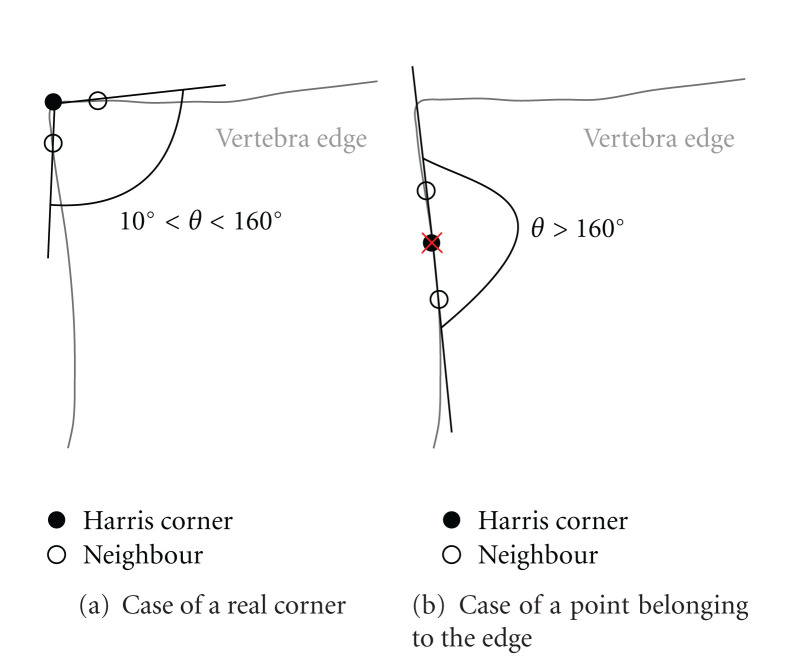
The angle filter process.

The step of angle filtering of false corners aims to eliminate the Harris corners belonging to an angle that are not similar to vertebra angles. The main idea is to compute for each assumed corner the angle formed by straight lines linking its neighbors (Figure 7). We consider a point as a corner if the angle is between 10° and 160°. This limitation may seem large, but takes into account some special cases: the vertebrae with a tip corner or a rounded one.

The distance between the corner and its neighboring points plays an important role. Indeed, if the neighbors are too close to the corner, the angle may be too straight and lead to a reject of the real corner. On the other hand, neighbors too far from corner may lead to the acceptance of too many false corners.

Figure 6(d) shows the effectiveness of this filter. It can eliminate 75% of the points and clean out the vertebrae of false corners.

### 2.4. Localization of the Vertebra Corners

The Harris corner detector provides a large set of points of interest. The previous filters reduce the number of elements on this set. The goal of this section is to determine exactly the 2*N* anterior corners of the *N* vertebrae among all the candidates.

To this end, we propose an algorithm based on the idea that looking for this sequence of 2*N* corners is equivalent to searching for the shortest path between the upper corner of the first vertebra and the lower corner of the last vertebra, composed by 2*N* points.

**Figure 8:**
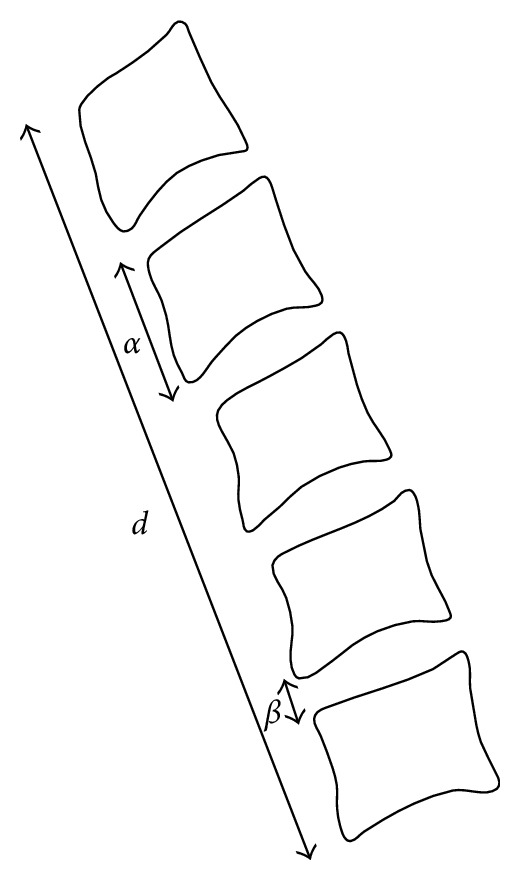
Parameters used for the vertebra corners detection.

In this kind of problem, the first idea—the simplest one—is to consider all the possible sequences between the upper anterior corner of the first vertebra and the lower anterior corner of the last one. To do so, we describe a procedure dedicated to the build of those sequences based on an initialization conducted by an operator. Let *l*_1_ be the upper anterior corner of the first vertebra and *l*_2*N*_ the lower anterior corner of the last vertebra. The user is given the task to mark out these two particular points. The first step of the algorithm consists in generating a first sequence *S*_1_ composed of the point *l*_1_. Next, all the points allowing to construct sequences of 2*N* points are considered. Nevertheless, such a method can turn out to be very time-consuming given the number of points previously detected. For this reason, an *“intelligent”* recursive function has been developed. Let *RecursiveFunction *(*S*_*i*_) be this function, where *S*_*i*_ is a sequence composed of *i* vertebra corners. *RecursiveFunction *(*S*_*i*_) is based on parameters about the cervical column. Let *α* be the height of a vertebra, *β* the size of an intervertebral space, and *d* the distance between the upper anterior corner of the first vertebra and the lower anterior corner of the last vertebra. These considerations are presented in Figure 8. Actually, we can approximate the relation between *d* and the parameters *α* and *β* by

(6) d≈Nα+(N−1)β,

where *N* is the total number of vertebrae.

Furthermore, practice gives us an empiric relation between *α* and *β*.

(7) α=4β.

We can therefore deduce

(8) $$\alpha =\frac{4}{5N-1}d.$$

Once all the parameters are determined, *RecursiveFunction *(*S*_*i*_) uses them in order to establish the list of the future points in the sequence. To explain the role of *RecursiveFunction *(*S*_*i*_) more precisely, let us consider a sequence *S*_*i*_ composed of *i* corners. The first step of the recursive function is to determine the type of the last point *l*_*i*_ in the sequence: upper or lower. Next, a set of acceptable points are considered based on the distance between them and the current point *l*_*i*_. If the point *l*_*i*_ is an upper one, the conditions to meet are given at (9). The criteria for a lower point are presented at (10). In both of these relations, the intervals represented by the variables *δ*_*α*_ and *δ*_*β*_ have to be fixed experimentally. Furthermore, the notation *dist*(*l*_*i*_, *l*) stands for the Euclidean distance between *l*_*i*_ and *l*:

(9) $$\begin{array}{c} \alpha -\delta_{\alpha }<dist\left(l_{i},l\right)<\alpha +\delta_{\alpha } \end{array},$$

(10) $$\begin{array}{c} \beta -\delta_{\beta }<dist\left(l_{i},l\right)<\beta +\delta_{\beta } \end{array}.$$

Every acceptable point is then added to the sequence *S*_*i*_. A recursive call to *RecursiveFunction *(*S*_*i*_) is made with the resulting sequence *S*_*i*+1_. For a matter of optimization, an additional constraint has to be reached: the acceptable points must have a *y*-coordinate lower than the one of the point *l*_*i*_.

Finally, the function stops when the number of points in the sequence is equal to 2*N* − 1. The point *l*_2*N*_ defined by the user is therefore added to the sequence. The latter is memorized in a set of sequences. Let *V* be this set. The function could also be stopped if there are no additional points respecting one of the conditions (9) and (10).

Once all the recursive calls are terminated, the function provided as a result a set *V* of sequences composed of 2*N* corners. For each sequence, the distance of the path between the upper corner of the first vertebra and the lower corner of the last vertebra is computed. The minimum of all these distances is extracted and defines the sequence of 2*N* corners retained for the initialization of the segmentation.

The global algorithm is given at Algorithm 1, and the recursive function is detailed at Algorithm 2.

**Figure 9:**
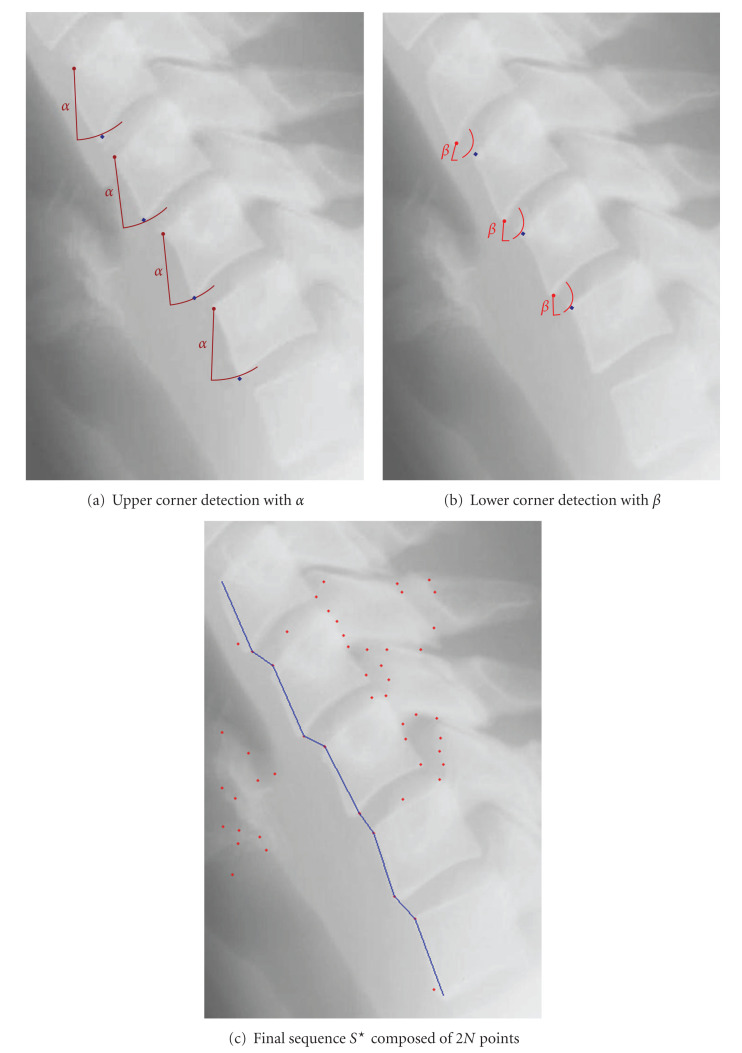
Illustration of the construction of the sequence *S*^⋆^.

In order to clarify the algorithm, we propose at Figure 9 an illustration of how the algorithm builds the optimal sequence *S*^⋆^ composed by the 2*N* vertebra corners.  Figure 9(a) illustrates the upper corner detection based on the parameter *α* while Figure 9(b) shows the lower corner detection based on the parameter *β*. The final sequence *S*^⋆^ is presented at Figure 9(c).

### 2.5. Segmentation

The previous steps allowed to determine the anterior corners position of every vertebra in the image. This way, it provides relevant information about the vertebra position, orientation, and height. Therefore, it becomes possible to precisely place the mean shape at every detected vertebra position in order to initialize the segmentation procedure.

**Figure 10:**
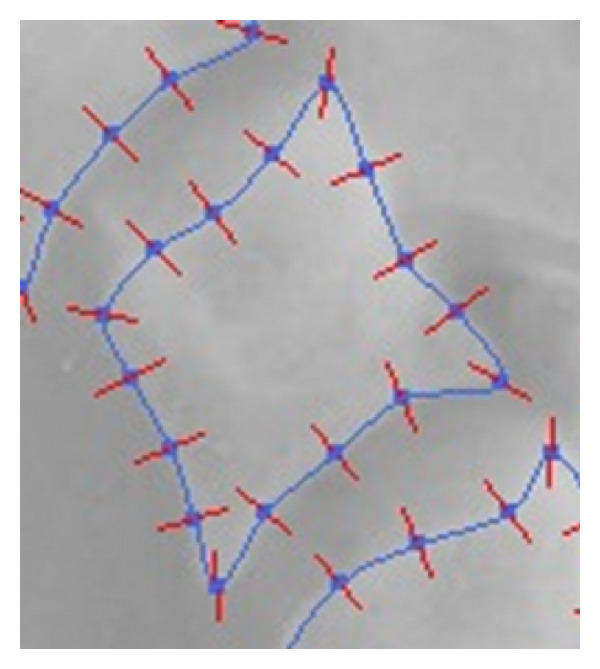
Normal of the contours at each point of the profile.

The ASM search treats every landmark defining the starting shape. For each of these points the neighborhood texture is analyzed in a specific direction. This analysis is made by considering landmarks along the normal of the contour at the considered point (see Figure 10). A profile *g* is then defined as a vector containing the gradient of intensity for each point in the normal. A landmark on the current shape is moved along the direction perpendicular to the contour, to the position where the profile is the closest to the mean shape ones according to the Mahalanobis distance [13]. This distance *D* is mathematically defined in

(11) $$D={\left(g-\overset{®}{g}\right)}^{T}S_{g}^{-1}\left(g-\overset{®}{g}\right).$$

In relation (11), the Mahalanobis distance gives a representation of the difference between a given profile *g* and the profile $\overset{®}{g}$ associated to the mean shape. *S*_*g*_ is a covariance matrix of the profiles related to the current landmark in the training set. In order to build *g*, we need to define some landmarks along the normal at the considered point. On each of these landmarks, the grey level (between 0 and 255) is evaluated. The gradient is obtained by subtracting the grey level for the point *i* with the grey level for the point *i* − 1 on the normal. Each value is finally normalized by the sum of each grey level in the profile.

All these considerations are detailed at Algorithm 3. In this algorithm, one can see that a convergence condition is used. Here, we propose to stop the search when all the landmarks of the shape remain stable, that is, do not change anymore. Nevertheless, it appears that this condition is too strict. Therefore, we compute the number of equivalent points that have a different position between the current and the previous shape. If we consider the iteration *i*, the search is stopped if the number of equivalent points with a different position between iteration *i* and iteration *i* − 1 is 10% the number of equivalent points with a different position between iteration *i* − 1 and iteration *i* − 2. In order to avoid infinite search loop, a maximum number of iterations can be defined. Generally, the convergence is reached after 50 to 250 iterations. To give an order of magnitude, the execution time for 50 iterations is about 15 seconds, based on our experimentations.

## 3. Results

Various parameters can significantly influence the results. In the following section, we propose investigating the influence of each of the following parameters:

- the number of landmarks per vertebra,
- the profile structure,
- the number of images used to build the sample,
- the mean shape model.

### 3.1. Number of Landmarks per Vertebra

**Figure 11:**
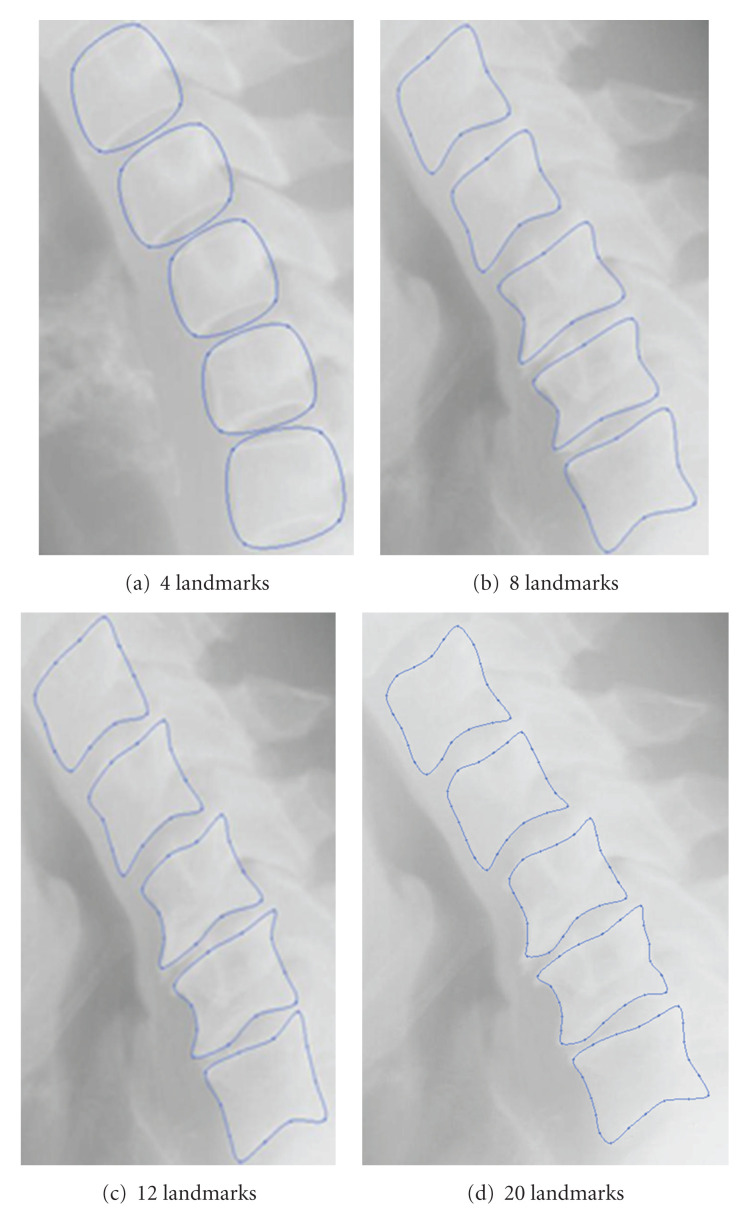
Influence of the number of landmarks.

The number of landmarks per vertebra (see Figure 11) has a direct influence on the quality of the segmentation results obtained by the search process. It is evident that the greater the number, the better the segmentation result. Nevertheless, it would be necessary to find a good compromise, in order to obtain a reasonable computing time for the search phase. In our experiments, 20 landmarks for each vertebra were used.

### 3.2. Profile Structure

The second parameter influencing the segmentation results is the structure of the profiles used for the search process phase. The profile depends on two other parameters: the number of points by profile and the distance between these points. We can notice that, in order to ensure the independence of this distance with respect to the image size, its length is proportional to the vertebra area. After various tests, we conclude that a profile of seven points spaced by a distance equal to 5% of the vertebra size is a good compromise.

### 3.3. Number of Images Involved in the Construction of the Sample

The size of the sample remains the most important aspect of the ASM method. It is the basis for building the statistical model of shape and determines the outcome of the segmentation, the final result in an instance of this model. The robustness of the method is reached only if the sample is as representative as possible of the data segmentation.

The specialist knowledge and the practitioner expertise can play a crucial role in the choice of the images for the sample.

In order to test this parameter, we used the single model of vertebra. The ASM search initialization is performed through the user intervention to mark the left side of the vertebrae C3 to C7 by clicking on the anterior superior and inferior corners.

**Figure 12:**
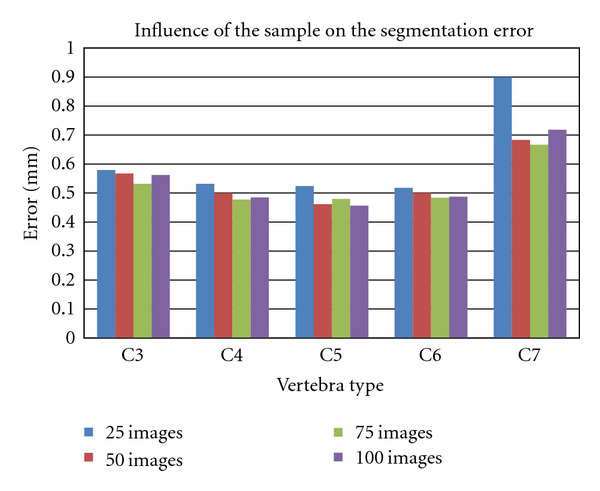
Influence of the sample size on the mean error of segmentation.

Figure 12 gives a statistical presentation of data and shows the influence of sample size on the average error of segmentation based on the type of vertebra. We use a sample size of 25, 50, 75, and 100 images, respectively.

**Figure 13:**
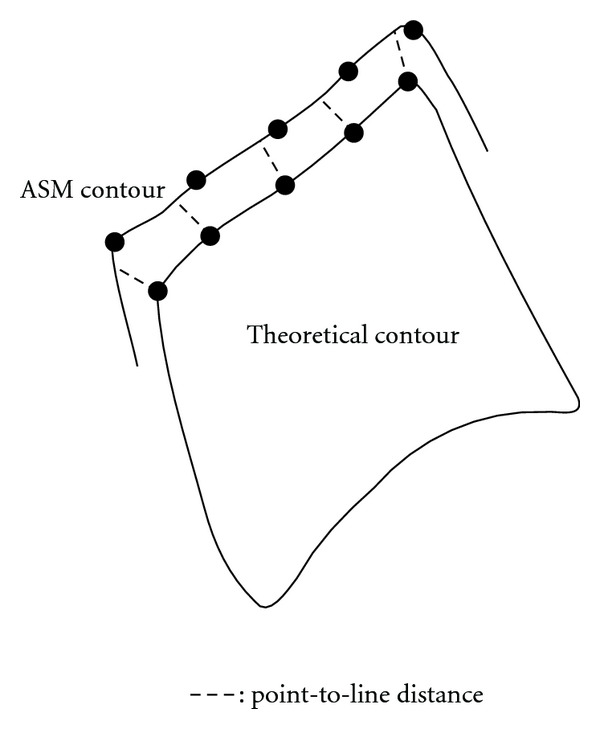
Point-to-line distance between 2 contours.

In order to represent the accuracy of the segmentation, we use a particular measure, that is, the point-to-line distance. An illustration of this distance is given at Figure 13. In fact, the vertebra edge is characterized by 20 landmarks, and a specialist is asked to mark the images of the database. This way, we create a gold standard (a theoretical contour) for the computation of the segmentation error. We calculate it by evaluating the distance between the segmented contour and the theoretical contour. Actually, the Euclidean distance is computed between equivalent landmarks on the segmented contour and the theoretical one. We perform this task on all the images in our database and report the mean error. Figure 12 shows that a sample size of 75 images is a good compromise for each vertebra. This study has allowed us to estimate the images number that could be involved in the construction of the sample.

### 3.4. Model

**Figure 14:**
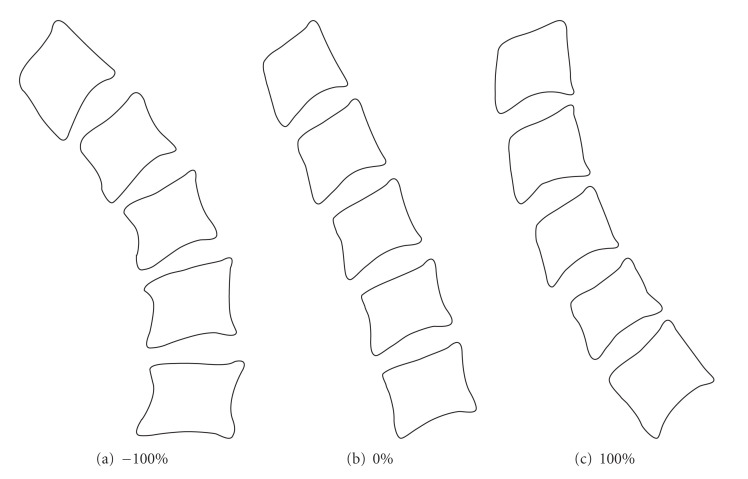
Effects of variations along the principal directions of a column mode.

We propose two models: the column model and the vertebra model. The first aims to describe all the vertebrae into a single form and thus contains the coordinates of their landmarks.  Figure 14(b) represents the average shape of a column model. Figures 14(a) and 14(c) show the shapes obtained by applying different displacements along the principal directions, within the limits provided by (5).

The main advantage of the column model is that it changes the whole column during the search process. A vertebra cannot be rotated independently of others. This can be useful to determine the curvature of spinal column. By consequence, this advantage becomes an obstacle for the detection of a vertebra different from others; hence isolating anomalies are more difficult.

The vertebra model consists of modeling every vertebra by only one model. Therefore, it allows to resolve the shortcomings of the global column model. It is also more suitable for local search in the image. Nevertheless, it has the disadvantage of ignoring information that exists between the vertebra shapes, since each of them can evolve independently.

**Figure 15:**
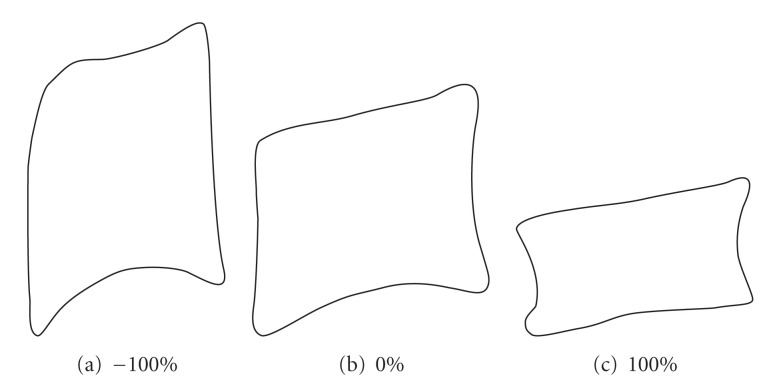
Effects of variations along the principal directions of a vertebra model.

Figure 15 shows the shapes obtained by applying the same movements according to the main direction of a vertebra model.

Table 1 proposes the vertebra recognition rate on 100 images from the online database NHANES II from the National Library of Medicine (NLM) [25]. It shows a comparison between the column model and the model of vertebra using a sample of 75 images. The NHANES II database is large enough to build separately the test set (100 images) and the training set (75 images) without performing a cross-validation. Furthermore, the test set is not included in the training set to avoid any influence. Given the results, our preference is for the vertebra model. Moreover, according to our experiments, the comparison between the results from both models shows that the edges of the vertebrae are well detected by using the model of a vertebra. More precisely, we consider that the vertebra model detects well the edges supported by the success rate (between 92.2% and 98%).

**Figure 16:**
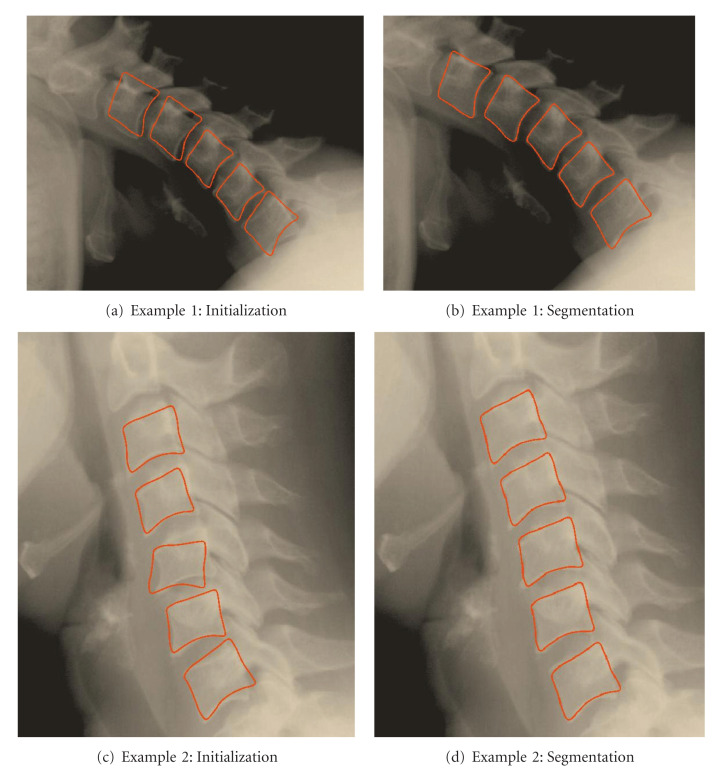
Results of segmentation using the vertebra model.

Figure 16 shows the segmentation results for some images corresponding to the cervical spinal column on the basis of the parameters presented above. After convergence, all the vertebrae are well detected. The segmentation results for the test images show that vertebra edges are detected by applying the proposed segmentation approach, based on a vertebra model and using the Active Shape Model approach.

Comparison with other approaches is quite difficult. The main reason is that the methods proposed in the literature are applied in different contexts. For instance, the imagery modality is not always the same or the type of vertebra is different. In [26], Roberts et al. applied the AAM segmentation approach on dual X-Ray absorptiometry images and obtained a mean error equal to 0.88 mm. They proposed a similar method based on AAM in [27] but applied on conventional radiographs. The mean error is equal to 0.64 mm. In our case, as Figure 12 shows, the segmentation error is about 0.6 mm. Nevertheless, we study cervical vertebrae while Roberts et al. studied lumbar vertebrae. In [28], de Bruijne and Nielsen presented a mean point-to-contour error of 1.4 mm using shape particle filtering. Finally, Klinder et al. obtained a mean point-to-surface segmentation error of 1.12 mm with CT images [29]. However, they ran experiments on every type of vertebra (cervical, lumbar, and thoracic).

## 4. Conclusion

 In this paper we presented a vertebra segmentation method using an Active Shape Model recognition approach. The Active Shape Model segmentation method is composed of two phases: a modeling phase, aiming to create a mean shape model, and a searching phase. An important challenge on applying this approach is the impact of the initialization, that is, the way that the mean shape model is placed on the image at the beginning of the search stage: the closer the mean shape is placed to the object, the better the chances of successful segmentation. In this paper, we solve this problem by using a semiautomatic segmentation process. Therefore, we suggest placing the mean shape model on the image by using the vertebra left corners, which are extracted in a semiautomatic way. This task was achieved by using the Harris corner detector and a set of successive filters, and only two points placed by the user (the superior left corner of the first vertebra and the inferior left of the last one). This approach produces an efficient initialization of the ASM search process. Additionally, we presented two modeling techniques: the vertebra model which consists in modeling vertebra shapes with only a single model and the column model which represents the whole shape of the spinal column.

Another inconvenient principal of the ASM-based segmentation approach is the training stage, for which we constructed the mean shape model by using, respectively, 50, 75, and 100 sample images. The choice of a sample of 75 images produces comparable results with the sample of 100 images.

In addition, we investigated the influence of various other significant parameters on the segmentation results. Thus, we studied the influence of the number of landmarks per vertebra, the profile structure, and the mean shape model. We concluded from this study that the best compromise is to choose 20 landmarks per vertebra and a profile structure of seven points. We also noticed that the results given by the vertebra model were more efficient than those given by the column model.

The various tests that we carried out on a large dataset prove the effectiveness of the suggested approach. We observe that the proposed method allows fast and efficient vertebra contours extraction. Our method can also be adapted to other components of the spinal column: like dorsal or lumbar. In our future works we want to investigate a method aiming to automate the segmentation. We consider also the use of the segmentation results to analyze the mobility of the cervical spinal column.

## Figures and Tables

**Algorithm 1:**
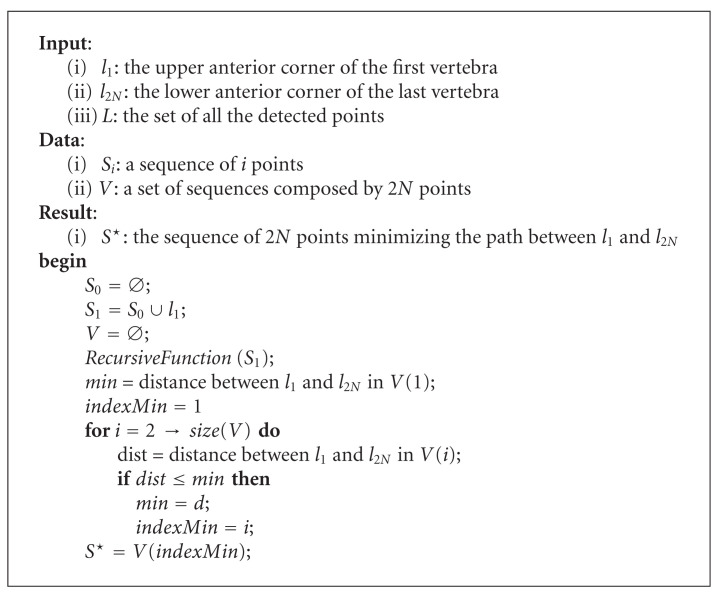
Determine the sequence of the vertebra corners.

**Algorithm 2:**
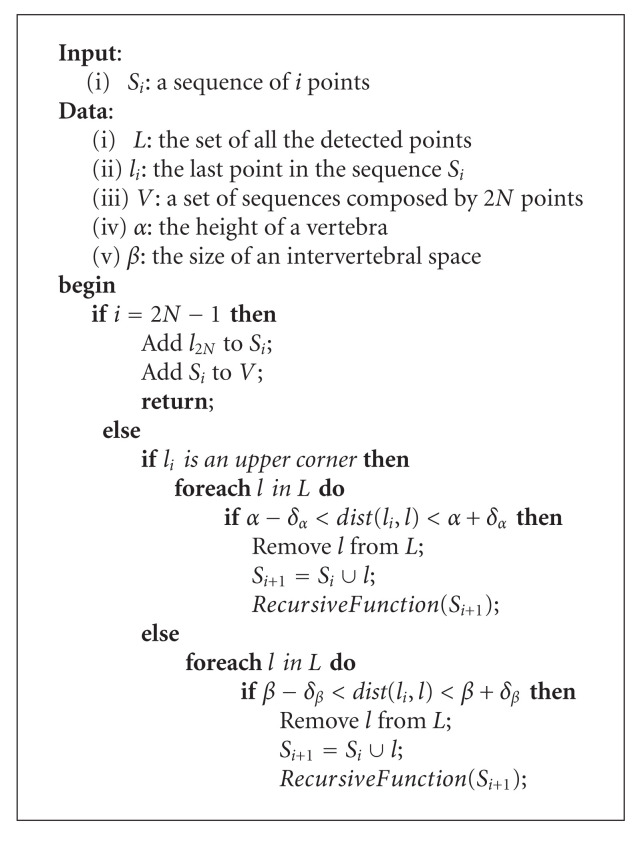
*RecursiveFunction *(*S*_*i*_).

**Algorithm 3:**
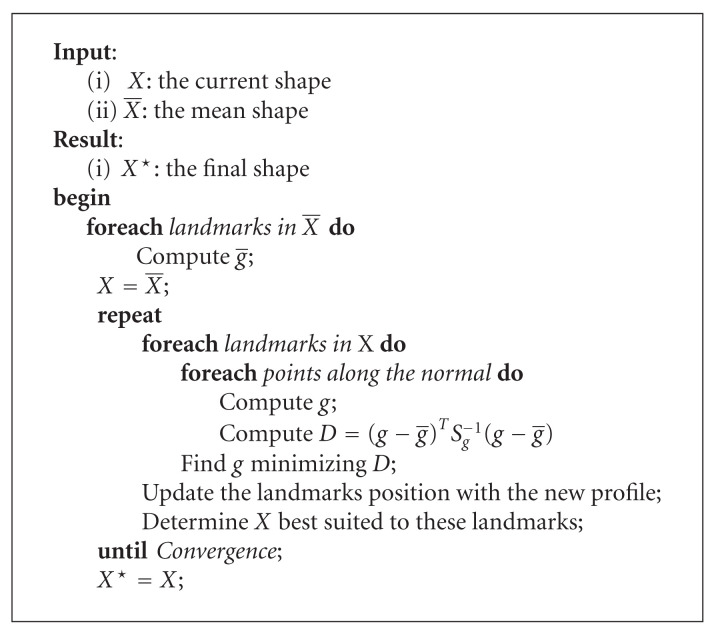
ASM segmentation procedure.

**Table 1:** Success rate of segmentation with a sample of 75 images.

	Sucess Rate (%)	
Type of Vertebra	Column Model	Vertebra Model
C3	54.9	92.2
C4	80.4	98
C5	82.4	96.1
C6	76.5	96.1
C7	56.9	98
